# The social cohesion investment: Communities that invested in integration programmes are showing greater social cohesion in the midst of the COVID‐19 pandemic

**DOI:** 10.1002/casp.2522

**Published:** 2021-04-05

**Authors:** Fanny Lalot, Dominic Abrams, Jo Broadwood, Kaya Davies Hayon, Isobel Platts‐Dunn

**Affiliations:** ^1^ Centre for the Study of Group Processes University of Kent Canterbury UK; ^2^ Belong—The Cohesion and Integration Network Manchester UK

**Keywords:** community psychology, COVID‐19, integrated communities, social cohesion

## Abstract

Social cohesion can rise in the aftermath of natural disasters or mass tragedies, but this ‘coming together’ is often short‐lived. The early stages of the COVID‐19 pandemic witnessed marked increases in kindness and social connection, but as months passed social tensions re‐emerged or grew anew. Thus local authorities faced persistent and evolving challenges. A cross‐sectional survey (*N* = 2,924) examined perceptions of social cohesion while Britain was slowly emerging from its first national lockdown in June 2020 in six English local authorities that have prioritised investment in social cohesion over the last two years (including five ‘integration areas’) compared with three other areas that have not. We expected that social cohesion programmes would better equip people to tackle the various challenges of the COVID‐19 pandemic. We found a greater sense of social cohesion in the six local authorities (at the *micro*, *meso* and *macro* levels) than in other areas. This was manifested as higher levels of reported social activism, interpersonal trust and closer personal relationships, greater political trust and more positive attitudes towards immigrants. Findings are consistent with the proposition that investing in social cohesion underpins stronger and more connected and open communities, better able to cope with crisis situations.

It is commonly expected that people come together in times of crisis, as global issues call for common and coordinated responses. For example, social cohesion and actions of solidarity have previously risen in the aftermath of financial crises (Borger, 2013), natural disasters (Calo‐Blanco, Kovářík, Mengel, & Romero, 2017) and mass tragedies (Hawdon & Ryan, 2011; see also Drury & Tekin Guyen, 2020). In the ongoing COVID‐19 pandemic, people have spontaneously organised in ‘mutual aid groups’ and other volunteering associations to support those most affected by the health crisis (Butler, 2020). However, this sense of ‘coming together’ is often short‐lived and social fragmentation may revert to pre‐disaster levels within a matter of weeks (Sweet, 1998; see also Hawdon & Ryan, 2011).

Indeed, a sense of shared social identity is needed to support collective solidarity (Ntontis & Rocha, 2020). In the early days of a crisis, the perception of a shared and global traumatising experience, combined with the necessity of common and coordinated responses, increases the perception of being ‘all in the same boat’ regardless of previous divisions between social groups (Drury, Brown, González, & Miranda, 2016; Muldoon, 2020)—facilitating collective solidarity. However, it is difficult for people or society to sustain a broadly inclusive level of self‐categorisation that overlooks meaningful intergroup differences (Abrams, Lalot, & Hogg, 2021). As a matter of fact, numerous xenophobic incidents against people of East Asian origin were reported worldwide (Nature Editorial, 2020), and in the United Kingdom, in the months following the first lockdown, a variety of sources of evidence suggested that social tensions were growing. Minority ethnic and religious communities have been accused of spreading the virus by not taking recommendations seriously (BBC News, 2020) as have younger people (Reicher, 2020)—potentially fuelling increased tensions between groups within and across local communities (see also Prosser, Judge, Bolderdijk, Blackwood, & Kurz, 2020).

As the impact of the pandemic continues to unfold, the challenges for communities are therefore persisting and evolving. As others have noted, group solidarity ‘is fragile and require long‐term investment’ (Ntontis & Rocha, 2020, p. 105). Social coordination has been essential for local areas to be able to implement local test, track and trace systems and to tailor health messages for different local groups and communities, and a degree of cohesion seems necessary in order to promote community resilience and help overcome future challenges (Hogan, 2020).

In this context, the present research project examines, in different parts of Britain, people's perception of social cohesion, specifically to compare areas that have explicitly invested in social cohesion programmes with areas that have not. Compared to people living in areas without such programmes, social cohesion programmes should better equip people to cope with the various challenges of the COVID‐19 pandemic, but this important proposition has yet to be tested empirically. In the next sections, we briefly review the literature on social cohesion and how to measure it, before turning to the comparative framework for the current research.

## 1 SOCIAL COHESION

Several definitions of social cohesion can be found in the literature and there is no clear consensus that a single rigorous definition can be used for empirical research (Bottoni, 2018a; Dickes, Valentova, & Borsenberger, 2010; see also The British Academy, 2019). The difficulty is at least partly due to the differing usage of the concept of social cohesion by institutions, policy makers and academics (Bottoni, 2018b; Chan, To, & Chan, 2006)—the latter also offering perspectives from a variety of fields (e.g., economics, political sciences, sociology and psychology). Although researchers generally agree that social cohesion is a multidimensional construct, they continue to debate the exact nature (and number) of these dimensions (for comprehensive discussions of different theoretical accounts, see, for example, Beauvais & Jenson, 2002; Chan et al., 2006; Fonseca, Lukosch, & Brazier, 2019; Schiefer & van der Noll, 2017).

In the present research, we draw from a recent theoretical multidimensional model of social cohesion (Bottoni, 2018a, 2018b), intended to be operationalisable in empirical research. Chan et al. (2006, p. 290) proposed the following definition of social cohesion: Social cohesion is ‘a state of affairs concerning both the vertical and the horizontal interactions among members of society as characterized by a set of attitudes and norms that includes trust, a sense of belonging and the willingness to participate and help, as well as their behavioural manifestations’. Bottoni (2018a, 2018b) then built upon earlier work by Whelan and Maître (2005) to distinguish between three levels of social cohesion:

- A *macro* level that reflects connections in, and sense of membership of, the broader society, and relations with institutions;
- A *meso* level that reflects connections with secondary groups (larger social in‐ and out‐ groups that can provide social identities);
- A *micro* level that reflects interpersonal connections with and trust in close others (mostly within families and between friends).

Bottoni also embraced Bollen and Hoyle (1990)'s contention that social cohesion has both a *subjective* perspective that focuses on people's perceptions (attitudes and state of mind), and an *objective* perspective that consider people's manifest behaviours (see also Chan et al., 2006). The resulting model of social cohesion was tested and supported through a cross‐cultural study involving 29 countries (data from the European Social Survey), which supported a multidimensional, three‐level, model of social cohesion across countries (Bottoni, 2018a, 2018b).

Seven key components emerge from Bottoni's integrative model, which capture the combinations of level (micro, meso, macro) and perspective (subjective, objective) features that underpin them, and which together comprise the higher‐order construct of social cohesion. Specifically, these are:

1. Institutional trust (macro/subjective)
2. Legitimacy of institutions (macro/objective)
3. Openness (meso/subjective)
4. Participation (meso/objective)
5. Interpersonal trust (micro/subjective)
6. Social support (micro/subjective)
7. Density of social relations (micro/objective)

## 2 INDICATORS OF SOCIAL COHESION

### 2.1 Macro level: Individual‐institutions relationships

*Institutional trust* or political trust refers to the confidence people have in their government, and the extent to which they see the government and attached institutions as trustworthy, credible, fair and competent (Levi & Stoker, 2000). At such, it represents a form of diffuse support for the authorities (Easton, 1975). Low levels of political trust pose a threat to social cohesion since they can result in tensions between citizens and their institutions (Bottoni, 2018b) and potential violence from both sides. For example, low trust is associated with increasing ‘noninstitutional engagement’, such as demonstrating (Kaase, 1999). Contrariwise, high levels of political trust are associated with increasing ‘institutional’ engagement, such as voting (Hooghe & Marien, 2013). They are also positively related to compliance with regulations and law‐abiding attitudes (Marien & Hooghe, 2011; Tyler, 2001).

*Legitimacy of institutions* is related to political trust. In systems that are not based on coercion, institutions need to be perceived as legitimate (i.e., strong reputation) to be able to exert their role (Bottoni, 2018b). Perceived legitimacy is best apprehended as citizens' level of satisfaction with the way things work in the country (e.g., state of education or health service), and with their national government.

### 2.2 Meso level: Individual‐group relationships

At the meso level, there are both attitudinal and behavioural features that reflect cohesion. *Openness* involves the acceptance of outsiders and outgroups. Positive relations between different groups in society are necessary for sustained social cohesion. Social psychology, and more specifically the social identity perspective, has established that people readily think of themselves as members of specific groups, which are different from other (out‐)groups (Abrams & Hogg, 2010; Tajfel & Turner, 1979). This self‐categorisation in terms of ‘us’ and ‘them’ can lead to ingroup favouritism and outgroup prejudice and discrimination, especially when outgroups are perceived as competing for limited resources or as representing a different and incompatible way of life with one's own (Stephan & Stephan, 2000; Stephan, Ybarra, & Morrison, 2009). Lack of contact with people from different groups also allows perpetuation or accentuation of antipathy towards outgroups (Pettigrew, Tropp, Wagner, & Christ, 2011). Contrariwise, in a cohesive society people will have more frequent contacts with others from different backgrounds (social, ethnic, etc.) and hold more positive attitudes towards them (Bottoni, 2018b). Consequently, many studies of social cohesion utilise immigration attitudes as an indicator of openness in the form of harmonious intergroup relationships.

*Participation* or engagement in social actions (Chan et al., 2006; Olson, 1971; van Zomeren, Postmes, & Spears, 2008) is the behavioural manifestation of cohesion at the meso level. Beyond holding positive attitudes, cohesion requires that people act on these attitudes and work actively and together towards a preferred state of things for everyone in society. Participation typically includes actions such as petition signing, joining an organisation or association, donating and volunteering.

### 2.3 Micro level: Relationships between individuals

Although less notable in policy discourse on social cohesion, social relations at the micro level (i.e., relations with primary social groups and close others) are considered a key component of social cohesion in several academic‐based frameworks (e.g., Berger‐Schmitt, 2002; Bottoni, 2018a; Chan et al., 2006; Duhaime, Searles, Usher, Myers, & Fréchette, 2004).

*Interpersonal trust* is a core component of social cohesion, necessary to sustain helping and cooperating behaviour with fellow members of society. Chan et al. (2006) propose that the importance of trust is ‘a quasi‐tautology, since it is virtually impossible to conceive of a situation in which we say people are “sticking” together even though they refuse to trust […] each other’ (p. 289). Research congruently shows higher levels of interpersonal trust (most often measured as agreement that ‘most people can be trusted’) are positively associated with social participation (e.g., volunteering; Anheier & Kendall, 2002; see also Ball et al., 2010).

*Social support*, related to trust, represents how much people feel appreciated, helped and supported by close others and people from their local area (Bottoni, 2018a). Social support is conceptually close to the concept of neighbourliness (or ‘good relations with the local area’; see Pilch, 2006) although the geographical boundaries are stricter for the latter.

*Density of social relations* taps into people's relationships with their closest others (often defined as friends, relatives or colleagues). It therefore represents social relations at the most immediate level. Strong and positive social relationships have long been identified as an important antecedent of personal well‐being and health (e.g., Cohen, 2004; Umberson & Karas Montez, 2010). A well‐established finding in the social psychology literature is that individuals have a need to belong to and be accepted by others (Baumeister & Leary, 1995; Festinger, 1953; Williams, 2009). In turn, individuals who feel accepted and supported are more likely to engage positively with and help others (Festinger, Schachter, & Back, 1950; Lott & Lott, 1969; see also Fonseca et al., 2019), triggering a virtuous circle of positive relations. Social relations hence form an important, final, component of social cohesion (Bottoni, 2018a, 2018b; Braaten, 1991).

## 3 THE ROLE OF PLACE AND THE UK GOVERNMENT INTEGRATION AREAS PROGRAMME

Studies of social cohesion have often identified differences in the level of cohesion between places, such as international differences (e.g., Bottoni, 2018b; Dickes et al., 2010) but also local‐based differences. Most importantly, differences at the local level in how the local government engages with and is perceived by the citizens, differences in local policies and programmes to foster positive intergroup relationships and contact, and different local socioeconomic factors, all seem likely to result in different levels of social cohesion. Therefore, a key aim of the present research was to investigate how the pre‐existence of local social cohesion programmes would affect levels of social cohesion during the COVID‐19 pandemic. To examine this question, we consider several local areas in the United Kingdom that had explicitly invested in social cohesion programmes over the past couple of years.

We now turn to the description of the UK context and these social cohesion programmes. The concept of ‘segregated lives’ and the need for ‘community cohesion’ arose in the United Kingdom following race riots in northern towns in 2001, and the publication of The Cantle Report (Cantle, 2001). From 2001 to 2010, UK community cohesion national policy and practice sought to increase cohesion between different (principally ethnic) communities to address segregation and far right sympathies in local areas. In 2010, policy moved towards a broader concept of ‘The Big Society’. Some limited social cohesion work was still supported via initiatives focused on young people and interfaith work but it was no longer prioritised. In 2016, Dame Louise Casey led a government review on integration and opportunities for the most isolated and deprived communities (Casey, 2016). The Casey Review brought issues of segregation and integration to the fore again, and in response the government published the Integrated Communities Strategy (2018) and the Integrated Communities Action Plan (HM Government, 2019; see also Donoghue & Bourke, 2019).

Amongst a range of initiatives, the Integrated Communities Action Plan funded five English local authority areas (Blackburn with Darwen, Bradford, Walsall, Waltham Forest and Peterborough) to serve as a test case that would develop local place‐based integration plans and programmes over a 2‐year period. Each local area developed a local strategy and programme of activities tailored to the issues impacting integration locally. During the two years that preceded the outbreak of COVID‐19, each area had hence faced different types of integration challenges and implemented programmes to strengthen social cohesion and integration locally. Broadly speaking, these programmes encompassed strengthening community leadership, supporting new migrants and local residents, education and young people, boosting non‐native speakers' proficiency in English language, focusing directly on place and communities, increasing economic opportunity and protecting rights and freedoms (HM Government, 2019). These programmes hence explicitly tapped into several core components of the social cohesion framework described earlier, most notably institutional trust, openness, participation, interpersonal trust and social support.

Along with any unifying effects, the COVID‐19 pandemic also placed obvious strains on social cohesion. As infection and death rates rose month after month, public and media attention turned more to finding culprits and assigning blame for the spread of the virus, fuelling intergroup tensions within and across local communities (Prosser et al., 2020; Reicher, 2020). Social distancing measures also impeded possibilities of meeting with close others, neighbours and people from the local area more broadly. In addition, the need for a political response and political guidance through the crisis led to increased attention to and scrutiny of political leadership, potentially fuelling political distrust and doubts on the legitimacy of the relevant institutions to implement valid and effective measures (Devine, Gaskell, Jennings, & Stoker, 2020).

Following elevated collective solidarity and cohesion in the early days of the crisis (Drury & Tekin Guyen, 2020; Ntontis & Rocha, 2020), subsequent increases in suspicion and decreases interpersonal contact, together with potential declines in trust in governing institutions and in other people all pointed towards a more dangerous longer term impact of the COVID‐19 as being social disintegration rather than cohesion. To the extent that these effects differ across places we may gain insight into not only where but also why some people and their communities are faring better, and ultimately to inform policy recommendations to foster locally‐based social cohesion more generally across periods of crisis.

## 4 OVERVIEW AND HYPOTHESES

The present data is part of a large‐scale research project designed to track social cohesion in the United Kingdom during COVID‐19. The research, conducted in partnership by the University of Kent and the charity Belong—The Cohesion and Integration Network, conducted surveys distributed online to respondents from different parts of Great Britain. An explicit goal of the research was to be able to compare social cohesion in places that have explicitly invested in social cohesion with the wider picture across other areas. The six investment areas therefore included the five local authority areas in the governments' Integrated Communities Action Plan. We also recruited participants from a sixth local authority (Calderdale) whose council, although not one of the government's integration areas, had prioritised kindness and resilience in its strategy over the last three years and which had also organised strongly community‐led responses to devastating local floods just prior to the pandemic (Calderdale Council, 2020). Calderdale Council has been in close contact with Belong and matched the inclusion criteria by having explicitly defined their vision for the locality to be ‘a place defined by our innate kindness and resilience, by how our people care for each other, are able to recover from setbacks and are full of hope’. These six local authorities together formed our ‘Integration and cohesion areas’ sample.

In order to provide a wider national comparison baseline, we also recruited participants living in the nations of Scotland and Wales as well as in the English county of Kent. These areas were chosen in part to represent differences in terms of demographics, political preferences and history, but also to gather a more nationally distributed and representative picture of levels of cohesion (e.g., across multiple districts and local authorities). By considering them together, we are able to establish a contextual comparison reflecting citizens' perceptions of social cohesion more generally in Britain. Analytically, we use them here as a control group, providing a baseline against which to contrast the levels of social cohesion observed in the integration and cohesion areas. Indeed, no systematic programme of social cohesion of the scale of the Integrated Communities Action Plan was implemented in these regions, allowing a direct comparison with the six integration and cohesion areas.^1^

We hypothesised that levels of social cohesion, measured through six indicators drawn from the literature (see above), would be higher in the six integration and cohesion areas as compared to the Other Places. A priori, because their strategies had been to build cohesion, we contend that where we observe differences these can, at least partly, be attributed to the integration and cohesion areas' active engagement in social cohesion programmes. However we also recognise that, despite the quasi‐experimental design, the evidence can at best support plausible explanation but cannot demonstrate causality.

## 5 THE PRESENT STUDY

We drew from Bottoni's social cohesion theoretical framework to consider six of the seven dimensions of social cohesion, two at each level of analysis (Bottoni, 2018a, 2018b)—namely: political trust, perceived legitimacy of institutions, attitudes towards immigrants (or openness), social participation, interpersonal trust and social relationships. When relevant, dimensions were measured with a specific focus on the pandemic; for example, perceived legitimacy of institutions was framed as satisfaction with the government's handling of the pandemic, and interpersonal trust was framed as trust in other people to respect the COVID‐19 restrictions in place. Detail of all measures is reported below.

In addition to these different dimensions, we considered two outcomes of social cohesion: subjective well‐being and optimism for the future. Indeed, research has consistently found that increases in social cohesion are related to increases in subjective well‐being (Delhey & Dragolov, 2016; Klein, 2013) and optimism (Brockie & Miller, 2017; Delhey & Dragolov, 2016). Well‐being and optimism are important variables to consider as they reflect the potential benefit of living in a more cohesive society for the self, effectively linking outcomes at the group and the individual level (Chuang, Chuang, & Yang, 2013).

Data were collected between 10 June and July 7, 2020 and are best considered in temporal context. A national lockdown had been initiated by the UK government on March 23, 2020. Lockdown rules were slightly relaxed on 10 May and some primary schools reopened in England on 1 June amidst much controversy. A 14‐day quarantine was imposed for all travellers to the United Kingdom on 8 June. The time window of data collection included the reopening of English retail shops and places of worship (15 June) as well as pubs and restaurants (4 July) following a lowering of the UK Alert level from L4 to L3 (19 June). This period also included reintroduction of local lockdowns, starting with the city of Leicester (30 June). Data collection concluded prior to further relaxation of quarantine rules for people arriving in the United Kingdom from abroad (10 July) and further easing of lockdown restrictions for England (announced 17 July; for a full timeline of events, see, for example, Aspinall, 2021).

### 5.1 Method

#### 5.1.1 Participants and procedure

Data were collected through an on‐line survey in which participants were recruited through external partners (Qualtrics Panels) and via social media and distribution through mailing lists by the partnering local councils. All participants were remunerated for their participation (£5, equivalent to €5.50 or $6). Sample size was determined prior to data collection based on feasibility and funding capacities (*n* = 200 in each of the integration and cohesion areas, *n* = 500 in each of Scotland, Wales and Kent). Surveys closed when the targeted sample size was achieved, or after 30 days elapsed (so as to keep the data collection period within one month). Only complete questionnaires were taken into consideration (across samples, dropout rates ranged 5–13%).

A total of 2,924 participants completed the survey (1,154 from the integration and cohesion areas and 1,770 from other areas). They were 1,142 male and 1,743 female (39 undisclosed) of a mean age of 51.2 years (*SD* = 15.6). All demographics (breakdown by Area) are reported in Table 1.^2^ Unless stated otherwise, all measures were assessed on 5‐point Likert scales. All descriptive statistics and reliability indices appear in Table 2 and correlations in Table 3. All item wordings and data are available on the OSF page dedicated to the project: https://osf.io/69cbn.

**TABLE 1:** Demographics breakdown

	Integration and cohesion areas		Other areas	
Demographic categories	Frequency	Percentage	Frequency	Percentage
*Gender*				
Male	352	30.5	790	44.6
Female	793	68.7	950	53.7
Undisclosed	9	0.8	30	1.7
*Age*				
18–24	65	5.6	75	4.2
25–34	159	13.8	197	11.1
35–44	262	22.7	293	16.6
45–54	241	20.9	293	16.6
55–64	235	20.3	385	21.7
65–74	152	13.2	395	22.3
75+	40	3.5	104	5.9
Undisclosed	0	0	28	1.6
*Ethnicity*				
White/white British	926	80.2	1,606	90.7
Asian/Asian British	109	9.5	29	1.6
Black/African/Caribbean/black British	28	2.4	8	0.5
Mixed/multiple ethnicity	21	1.8	14	0.8
Other ethnicity	21	1.8	2	0.1
Undisclosed	49	4.3	111	6.3
*Annual household income*				
Less than £15,000	135	11.7	231	13.1
£15,000 to £30,000	285	24.7	438	24.8
£30,000 to £40,000	173	15.0	284	16.0
£40,000 to £60,000	221	19.2	296	16.7
£60,000 to £100,000	138	12.0	172	9.7
More than £100,000	31	2.7	44	2.5
Undisclosed	171	14.8	305	17.2
*Political orientation*				
Left‐wing	489	42.4	561	31.7
Centre	460	39.8	675	38.1
Right‐wing	198	17.2	528	29.9
Undisclosed	7	0.6	6	0.3
*Subjective socio‐economic status*				
Mean (*SD*)	4.46 (1.27)		4.36 (1.31)	
Total	1,154	100	1770	100

*Note*: Political orientation is measured on a 7‐point scale (1 = Left‐wing, 4 = Centre, 7 = Right wing). For the table breakout we considered 1–3 as left‐wing, 4 as centre, 5–7 as right‐wing. In the analyses, however, the variable is kept continuous. Subjective socio‐economic status is measured on a 8‐point scale (status ladder), a higher rung (higher score) representing a higher subjective status.

**TABLE 2:** Descriptive statistics and reliability indices of social cohesion measures

Construct	Nb of items	*M* (*SD*)	α/ω_*t*_
Political trust	3	2.63 (0.81)	0.83/0.88
Appropriateness of restrictions	2	2.48 (0.91)	0.65/0.65
Attitudes towards immigrants	4	53.4 (22.5)	0.74/0.74
Social activism	14	2.08 (2.31)	–
Trust in others to respect restrictions	5	2.81 (0.70)	0.78/0.85
Personal relationships during lockdown	5	2.70 (0.78)	0.71/0.82
Subjective well‐being	2	3.55 (0.92)	0.91/0.91
Optimism	2	3.24 (0.99)	0.85/0.85

*Note*: Attitudes towards immigrants were measured with 2 × 2 items distributed randomly across participants (i.e., each participant rated 2 items only). These items took the form of a 100‐point feeling thermometer. Social activism is a summed score of up to 14 possible collective actions (1 = has done, 0 = not done). All other constructs were measured on 5‐point scales (average scores).

**TABLE 3:** Correlation matrix (Pearson's coefficients, zero‐order) between social cohesion measures and area

	Construct	1	2	3	4	5	6	7	8
1	Political trust								
2	Appropriateness of restrictions	0.17***							
3	Attitudes towards immigrants	0.11***	−0.14***						
4	Social activism	−0.00	−0.11***	0.25***					
5	Trust in others to respect restrictions	0.26***	0.29***	0.13***	0.03				
6	Personal relationships during lockdown	0.03	0.02	0.10***	0.05**	0.06**			
7	Subjective well‐being	0.18***	0.11***	0.07***	0.02	0.22***	0.09***		
8	Optimism	0.18***	0.18***	0.03	0.05**	0.25***	0.05*	0.48***	
9	Area (*1 = Integration and cohesion areas, −1 = Others*)	0.04*	0.04*	0.15***	0.27***	0.08***	0.13***	0.04*	0.08***

* *p* < .05.

** *p* < .01.

*** *p* < .001.

#### 5.1.2 Materials

##### Macro level

*Political trust*, the first indicator of social cohesion at the macro level was measured with 3 items (e.g., ‘Politicians are mainly in politics for their own benefit and not for the benefit of the community’, 1 = Strongly disagree, 5 = Strongly agree).

Perceived legitimacy of institutions was assessed through the *perceived appropriateness* of the current COVID‐19 governmental restrictions (2 items, for example ‘Do you think the UK government guidelines in place right now are too relaxed or too strict?’, 1 = Much too relaxed, 3 = About right, 5 = Much too strict).

##### Meso level

*Social activism* was measured by asking participants to indicate how many of 14 collective actions they had engaged in during the past month (e.g., signing a petition, demonstrating, donating, volunteering). We summed the numbers of activities reported to obtain an index (score from 0 to 14). Overall, 67% of participants had engaged in at least one activity during the past month.^3^

Openness was measured by assessing *immigration attitudes* with a 100‐point feeling thermometer (higher score representing ‘warmer’ attitudes; see, for example Wittenbrink, Judd, & Park, 2001) towards four groups (e.g., ‘Legal immigrants’, 0 = Very cold feelings, 50 = Neither warm nor cold, 100 = Very warm feelings).^4^

##### Micro level

Interpersonal trust was measured through questions about *trust in others to respect the COVID‐19 restrictions* (5 items, for example ‘How much do you think people from each group can be trusted to follow the government instructions about social distancing?—People in the United Kingdom in general’, 1 = Not at all, 5 = Completely).

Social relationships were assessed as change in *personal relationships during lockdown* (5 items, for example, ‘How would you say your connection with your family has changed during lockdown?’, 1 = Much less connected than usual, 5 = Much more connected than usual).

##### Outcomes of social cohesion

Two outcomes of social cohesion were assessed: *subjective well‐being* (2 items, for example ‘All things considered, how satisfied are you with your life as a whole nowadays?’) and *optimism* (2 items, for example ‘In uncertain times, I usually expect the best’, 1 = Not at all like me, 5 = Very much like me).

## 6 RESULTS

### 6.1 Analytical strategy

We used structural equation modelling first to evaluate the adequacy of the 3‐dimensional model to measure cohesion, and secondly to assess differences in social cohesion between the integration and cohesion areas and other areas (1 = Integration and cohesion areas, −1 = Other areas). SEM analyses indeed allow testing of interrelationships between a range of variables simultaneously. Analyses were conducted in *R* with the *lavaan* package (Rosseel, 2012) and used case‐wise (or ‘full information’) maximum likelihood estimation, including the measurement model (i.e., definition of the latent variables) and the structural model.

Prior to testing our specific hypotheses, we investigated the theoretical 3‐dimensional model of social cohesion. SEM results supported the idea that the six indicators of social cohesion were organised around three dimensions, which together contributed to a higher‐order construct of social cohesion. Results are reported in a note.^5^

We then moved on to the test of our hypotheses. We controlled for the following demographics: age (standardised), gender (1 = Female, −1 = Male), ethnicity (1 = White, −1 = Other than White), income (standardised), subjective socio‐economic status (standardised) and political orientation (standardised). The model tested the links between area (and all demographics) and the different indicators of social cohesion and in turn the links between these indicators and the outcome variables (subjective well‐being and optimism). We relied on suggested modification indices to allow pairs of items to co‐vary, when doing so significantly improved the model fit. Only pairs or close items (i.e., items contributing to the same latent variable) were allowed to co‐vary (see details in Supplementary Material SM2).^6^

To assess the model fit, we adopted a ‘2‐index presentation strategy’ in order to minimise both Type I and Type II errors (Hu & Bentler, 1999) and reported root mean square error of approximation (RMSEA; Steiger & Lind, 1980; see also Diamantopoulos & Siguaw, 2000) and standardised root mean residual (SRMR; Bentler, 1995). We also report comparative fit index (CFI; Bentler, 1990) and chi‐square. Typically, CFI ≥ 0.90, RMSEA ≤ 0.08, and SRMR ≤ 0.09 indicate an acceptable fit (MacCallum, Browne, & Sugawara, 1996).

### 6.2 Structural equation modelling results

**FIGURE 1:**
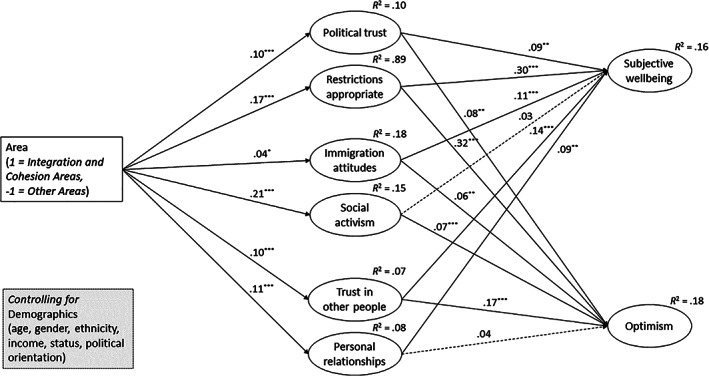
Structural equation model testing the effect of area (and demographics) on indicators of social cohesion; and the effect of these indicators on subjective well‐being and optimism. *Note*: Numbers reported are standardised coefficients. Dashed lines represent nonsignificant paths. Explained variance of each construct is reported (*R* ^2^). See Supplementary Material (SM3) for the effects of all demographics (included as covariates)

Results are illustrated in Figure 1 and mean differences between integration and cohesion areas and other areas are reported in Table 4. The complete inputs including all effects of demographics can be found in Supplementary Material (SM3). Overall, the model showed good fit indices, χ^2^(272) = 2076, CFI = 0.911, RMSEA = 0.048, 90% CI [0.046, 0.050], SRMR = 0.049. Consistent with our hypothesis, integration and cohesion areas showed higher levels of social cohesion on all indices. Specifically, respondents from integration and cohesion areas had higher political trust, perceived the COVID‐19 government restrictions as more appropriate, had more positive attitudes towards immigrants, had engaged in more social actions during the past month, had greater trust in other people to respect COVID‐19 government restrictions and finally reported greater personal relationships during lockdown than respondents from other areas. These measures of social cohesion, in turn, were related to higher subjective well‐being and greater optimism for the future (explaining 16% and 18% of variance, respectively).

**TABLE 4:** Effect of area (integration and cohesion areas vs. other areas) on the different indices of social cohesion

	Other areas *M* (*SE*)^a^	Integration areas *M* (*SE*)^a^	*b* (*SE*)	*z*‐test	*p*‐value	Cohen's *d* (variance of *d*)
*Macro level*						
Political trust	2.60 (0.021)	2.68 (0.027)	0.065 (0.017)	3.90	< .001	0.098 (0.002)
Appropriateness of restrictions	2.39 (0.023)	2.58 (0.029)	0.045 (0.009)	5.00	< .001	0.214 (0.002)
*Meso level*						
Immigration attitudes	52.8 (0.544)	55.0 (0.698)	0.977 (0.409)	2.49	.017	0.104 (0.002)
Social activism	1.68 (0.056)	2.73 (0.072)	0.492 (0.043)	11.52	< .001	0.482 (0.002)
*Micro level*						
Trust in others to respect restrictions	2.77 (0.018)	2.90 (0.023)	0.053 (0.018)	2.95	.003	0.186 (0.002)
Personal relationships during lockdown	2.63 (0.020)	2.79 (0.026)	0.091 (0.020)	4.54	< .001	0.218 (0.002)

^a^ Means are estimated marginal means, controlling for demographics (age, gender, ethnicity, income, subjective socio‐economic status and political orientation).

Additional analyses showed that area was linked to differences in optimism, so that respondents from integration and cohesion areas reported higher optimism, *b* = 0.074, *SE* = 0.017, *z*‐test = 4.50, *p* < .001 (*M* = 3.35, *SE* = 0.032, versus *M* = 3.18, *SE* = 0.025). Differences in well‐being did not reach significance, *b* = 0.032, *SE* = 0.018, *z*‐test = 1.80, *p* = .072 (integration and cohesion areas: *M* = 3.61, *SE* = 0.029; other areas: *M* = 3.55, *SE* = 0.023).

The greatest difference between integration and cohesion areas and other areas was found for engagement in social activism. Differences in trust in other people, personal relationships, political trust and perception of restrictions as appropriate, were of small effect size. Differences in immigration attitudes were of very small size.

### 6.3 Similarities and differences within the six integration and cohesion areas

Although all six local authorities had invested in social cohesion, it is important to note that each employed different strategies for doing so. This leaves open the possibility that some may have had stronger impact than others. Sensitive to this possibility, we explored the similarities and differences in social cohesion reported amongst the six integration and cohesion areas. A series of ANOVAs (again controlling for all demographics) revealed significant albeit small differences between the areas on most social cohesion indices (political trust: *F*(5, 931) = 2.38, *p* = .037, *η* ^2^_*p*_ = 0.013; appropriateness of government restrictions: *F*(5, 931) = 2.08, *p* = .066, *η* ^2^_*p*_ = 0.011; immigration attitudes: *F*(5, 931) = 3.98, *p* = .001, *η* ^2^_*p*_ = 0.021; social activism: *F*(5, 931) = 17.0, *p* < .001, *η* ^2^_*p*_ = 0.085; trust in others: *F*(5, 931) = 2.39, *p* = .036, *η* ^2^_*p*_ = 0.013; personal relationships: *F*(5, 930) = 3.17, *p* = .008, *η* ^2^_*p*_ = 0.017) as well as on its outcomes (well‐being: *F*(5, 931) = 2.79, *p* = .017, *η* ^2^_*p*_ = 0.015; optimism: *F*(5, 931) = 3.53, *p* = .004, *η* ^2^_*p*_ = 0.019). A careful investigation revealed that most of these differences were attributable to one of the integration and cohesion areas showing lower scores on the indicators considered than the five others. Re‐running the comparative ANOVA analysis without this specific area showed much homogeneity amongst the five others. Specifically, these five areas did not differ significantly in levels of political trust, *F*(4, 783) = 1.85, *p* = .12, *η* ^2^_*p*_ = 0.009, appropriateness of the restrictions, *F*(4, 783) = 1.12, *p* = .34, *η* ^2^_*p*_ = 0.006, immigration attitudes, *F*(4, 783) = 0.47, *p* = .076, *η* ^2^_*p*_ = 0.002 and trust in others, *F*(4, 783) = 1.21, *p* = .31, *η* ^2^_*p*_ = 0.006. Small differences did still exist in personal relationships, *F*(4, 782) = 3.38, *p* = .009, *η* ^2^_*p*_ = 0.017, social activism, *F*(4, 783) = 5.41, *p* < .001, *η* ^2^_*p*_ = 0.027, well‐being, *F*(4, 783) = 3.22, *p* = .012, *η* ^2^_*p*_ = 0.016 and optimism, *F*(4, 783) = 2.65, *p* = .032, *η* ^2^_*p*_ = 0.013. However, the important point is that each of the six integration and cohesion areas considered independently (even the one with least positive indicators) showed greater social cohesion across indicators than the other areas.^7^

A similar analysis to compare the three areas comprising other areas also showed much similarity between the different samples, with no significant difference arising on political trust, *F*(2, 1,485) = 0.002, *p* = .99, *η* ^2^_*p*_ = 0.000, social activism, *F*(2, 1,485) = 0.58, *p* = .56, *η* ^2^_*p*_ = 0.001, trust in others, *F*(2, 1,485) = 0.33, *p* = .72, *η* ^2^_*p*_ = 0.000, personal relationships, *F*(2, 1,485) = 0.43, *p* = .65, *η* ^2^_*p*_ = 0.001 or optimism, *F*(2, 1,485) = 2.45, *p* = .086, *η* ^2^_*p*_ = 0.003. Small differences arose for immigration attitudes, *F*(2, 1,485) = 4.00, *p* = .019, *η* ^2^_*p*_ = 0.005, appropriateness of the guidelines, *F*(2, 1,485) = 10.6, *p* < .001, *η* ^2^_*p*_ = 0.014 and well‐being, *F*(2, 1,485) = 4.02, *p* = .018, *η* ^2^_*p*_ = 0.005.

Finally, conscious that all six local authorities are in England, and that one might question the comparability with places in Wales or Scotland, we conducted a MANOVA, directly comparing these only with the Kent (i.e., English) respondents from the other areas. Results confirmed a higher level of social cohesion in the local authorities, *F*(6, 1,395) = 15.12, *p* < .001, *η* ^2^_*p*_ = 0.061.

In summary, there was greater similarity *within* each category of area (i.e., within integration and cohesion areas and within other areas) than *between* them. Those differences that did emerge within the categories were small in size, and were mostly related to one specific integration and cohesion area. Means for each subsample are reported in Supplementary Material (SM4).

## 7 DISCUSSION

Social cohesion may be crucial in times of crisis if local communities are to be able to implement efficient actions, promote community resilience and help overcome future challenges. Although social cohesion often ‘naturally’ increases when a community faces a crisis (Calo‐Blanco et al., 2017; Drury et al., 2016; Hawdon & Ryan, 2011), this initial sense of coming together can also fade rapidly (Sweet, 1998). The policy and practical challenge is hence to develop and maintain social cohesion in the longer run, especially when the crisis persists, such as with the ongoing COVID‐19 pandemic (Ntontis & Rocha, 2020). In the present research, we compared social cohesion in different parts of Britain and investigated whether areas that had invested in social cohesion through integrated community programmes were faring better than areas that had not. We proposed that these programmes should have equipped the community with useful social tools that could prepare them better to face difficult times. We predicted that these areas should show greater sustained social cohesion during the pandemic than areas that had not implemented such programmes.

The results supported our hypothesis. Controlling for relevant demographics, participants from five English integration areas and another that had invested in a cohesion strategy reported a higher sense of social cohesion measured through six indicators adapted from the literature. They showed greater political trust, more positive perception of government restrictions related to the pandemic, greater social activism, more positive immigration attitudes, greater trust in other people to respect the government restrictions and better personal relationships during lockdown than participants from areas that did not benefit from cohesion programmes. More positive levels on these dimensions of social cohesion were in turn related to greater subjective well‐being and optimism for the future, highlighting the benefits of socially cohesive places not only for the broad community but also for the individuals within them.

The beneficial outcomes of the cohesion and integration programmes were particularly visible in terms of engagement in social activism, trust in other people, personal relationships, and perception of governmental restrictions as appropriate. Further analyses demonstrated much homogeneity in the levels of social cohesion reported between the different integration and cohesion areas although one area showed lower levels of social cohesion than the others.

We believe it is reasonable to infer that the social cohesion programmes sustained social cohesion most clearly at the *meso* and the *micro* level (i.e., relations with secondary groups, and relations with primary groups and close others). This seems to align with the broad vision of the Integrated Communities Strategy, which aimed to act at the most local level of place and community (HM Government, 2019). Specific objectives such as ‘supporting and integrating new migrants’, or ‘strengthening leadership’ also seem to have been impacted by the integration programmes, although the effect sizes revealed by the present data are more modest. It is important to note, however, that respondents in the present survey were solely selected on the basis of where they lived, regardless of whether they had personally been involved in the integration programmes and even regardless of their knowledge of the existence of such programmes. Hence, we believe that identifying these differences in social cohesion is of substantive importance even if effect sizes are smaller.

The capacity to sustain social cohesion seems likely to have important immediate implications for individuals and their communities during the COVID‐19 pandemic. For example, people who feel greater political trust and who perceive governmental restrictions to be appropriate are more likely to adopt the health protective behaviour (mask wearing, social distance measures, etc., see Devine et al., 2020). Trust is also associated with greater prosocial behaviour during the pandemic (helping others financially and psychologically even at one's own expenses, Han et al., 2020). Stronger personal relationships are associated with a more positive experience of the first lockdown and lessened dread of future lockdowns (Fancourt, Bu, Mak, & Steptoe, 2020). Hence, it can be expected from the present evidence that the integration and cohesion areas went through a lesser hardship during 2020 than would otherwise have been the case had they not previously invested in social cohesion. An important empirical contribution of the present evidence is to show that, by contextualising the measures appropriately, social cohesion can be measured in relation to specific contexts or challenges such as the ongoing pandemic.

The present research also has theoretical implications. Notably, we have tested and supported the general proposition that cohesion can act as a societal buffer that may sustain communities and individuals. Our empirical test of the multidimensional model of social cohesion put forward by Bottoni (2018a, 2018b) supports the contention that indicators of social cohesion can usefully be distinguished at the *macro*, *meso* and *micro* level. The evidence also fits with a social identity theory perspective that would expect place (here operationalised as local area) represents a meaningful social category around which cohesion can be organised.

### 7.1 Limitations and future directions

Some limitations to the present study must be recognised. First, the cross‐sectional design of the research limits a causal interpretation of the results, notably on the question of whether social cohesion *leads to* greater well‐being and optimism. Nonetheless, it seems more plausible that place‐related differences in well‐being are affected more by the external conditions than by individual differences in temperament or personality. Longitudinal studies would be helpful to assess the changing aspect of social cohesion in times of crisis across places. Second, the method of distribution of the survey (i.e., in part through social media ads and mailing lists) means we cannot exclude some degree of selection bias in the individuals who decided to answer. Notably, female respondents were overrepresented in the integration and cohesion areas samples. Accounting for demographics in the analyses should have mitigated the impact of potential selection biases, but it remains possible that other non‐demographic factors led to sampling biases. Whilst it would be useful to compare the present evidence with data from larger representative samples in order to ensure their wider validity, we are unaware that any comparable data are available at the level of local authorities. Finally, the presence of differences amongst different integration and cohesion areas point to interesting avenues for future exploration. For example, the specificities of each local programme might strengthen particular dimensions of social cohesion more than others. Regardless of these nuances, across the different samples in our surveys, the evidence does speak to the positive connections between cohesion on the one hand and individual well‐being on the other. It remains for future research, local government and stakeholders to evaluate in more detail how elements of any particular programme may yield specific outcomes over time.

## 8 CONCLUSIONS

The present evidence is the first to test and support the contention that local cohesion programmes are playing an effective role in supporting social cohesion in the face of the COVID‐19 pandemic. It is worth noting that the six integration and cohesion areas were initially recruited for action because of their dire situation and the important community challenges they faced in 2018. It is therefore even more remarkable that two years later in the context of this pandemic, they appear to fare *better* than other areas in Britain, which, on average, arguably faced less severe challenges. Hence, the present findings are consistent with the expectation that investing in social cohesion can be a significant vehicle for building stronger, more connected and resilient communities that are better able to cope with crisis situations and more welcoming and open towards others (Brockie & Miller, 2017). This conclusion would support calls for further investment in integration and social cohesion (Callaghan & Colton, 2008; Chaskin, 2008; López‐Marrero & Tschakert, 2011).

## Supporting information

Supporting Information

## Data Availability

All item wordings and data are available on the OSF page dedicated to the project: https://osf.io/69cbn. Code is available in Supplementary Material.

## Endnotes

^1^
All areas of the United Kingdom address social differences in various ways but these tend to be piecemeal rather than in the form of whole area cohesion strategies. This was also true across Kent, Scotland and Wales, where their scope was generally not focused on the societal and psychological aspects of social cohesion. For example, a number of initiatives in Scotland aimed to redress economic inequalities (e.g, innovation and business competitiveness funding) and those categorised as ‘social inclusion’ initiatives were most often targeting poverty reduction specifically (Scottish Government, 2020). Similarly, the Welsh Government issued Community cohesion national delivery plans (the most recent two spanning 2014–2016 and 2016–2017) mostly focused on the issues of poverty, hate crime and violence (Welsh Government, 2014, 2015, 2016). The only outcome listed in the 2016–2017 national delivery plan explicitly mentioning social cohesion was limited to the Well‐being of Future Generations Act 2015, which broadly focuses on sustainable development – social cohesion being just one of seven well‐being goals defined there. Finally, in Kent we found no county‐wide social cohesion programmes. One County Council committee addresses loneliness and social isolation, primarily amongst older residents, but does not address social cohesion in the broader sense (Kent County Council, 2021). We noted that Kent is part of The Linking Network's schools linking national programme (Shannahan, 2018, p. 4), which aims to ‘enable children and young people to explore identity, celebrate diversity, build community and champion equality through the development of mutual understanding, critical awareness and openness, empathy, respect for difference and active citizenship’. This is directed at children aged 7 to 10 (school year 3–4‐5). It is possible that the programme translates to improved social cohesion with time as these children grow older, but it would have only limited impact amongst the adult samples assessed in the present research.

^2^
For information purposes, we report the complete demographic breakdown and comparison with official census data in Supplementary Material (SM1). Comparisons show that the different samples are roughly representative in terms of age and ethnicity, but gender cannot be considered representative (with an overrepresentation of female in several samples). We want to highlight however that the research project made no claim about the overall representativeness of the samples, owing to the difficulty of recruiting participant in niche locations such as the integration areas. For this reason, all analyses are controlled for demographics so that any difference observed cannot be imputed to underlying differences in demographics.

^3^
It could be argued that the summed score of number of actions might not completely reflect the intensity of social activism because it does not capture whether people have been engaging in these actions for a longer or shorter time. However, the purpose of this measure is to assess social activism in the context of the ongoing pandemic, that is in a specific and shorter time window and within the one‐month time window it seems reasonable to conclude that a person who engaged in a larger number of types of actions within the same time window was also more actively involved than a person who engaged in fewer types of actions (similar considerations affect the intergroup contact literature where quantitative and qualitative elements of contact can be linked to different factors but tend to be highly related to one another within relatively short time periods; see for example Hässler et al., 2020).

^4^
Attitudes were also measured towards some other social groups, which are beyond the scope of the present paper. Because of space constraints we used random assignment to ask participants rated only two of four different categories of immigrants: ‘Asylum seekers’, ‘Seasonal workers (migrant labour)’, ‘Legal immigrants’ and ‘Illegal immigrants’. Respondents from the integration areas and other areas were equally distributed across the random allocation, χ^2^(1) = 0.22, *p* = .64.

^5^
We tested the hypothesised 3‐dimensional model of social cohesion on the entire sample (Integration areas and Other areas). All indicators loaded on the expected dimension (either macro, meso or micro), and the three dimensions were significantly intercorrelated; this model showed a good fit, χ^2^(93) = 716, CFI = 0.934, RMSEA = 0.048, 90% CI [0.045, 0.051], SRMR = 0.043. In comparison, a unidimensional model provided a worse fit, χ^2^(96) = 977, CFI = .911, RMSEA = 0.056, 90% CI [0.053, 0.059], SRMR = 0.048 (Δχ^2^(Δ_df_ = 3) = 261, *p* < .001).

^6^
For information purposes, the fit of the original model (before adding covariances between pairs of items) was: χ^2^(283) = 3,888, CFI = 0.823, RMSEA = 0.066, 90% CI [0.064, 0.068], SRMR = 0.063. We then modified the model based on modification indices. We initially added five covariances between personal relationships’ items (modifications indices: neighbours/local = 837, family/friends = 623, friends/colleagues = 223, friends/neighbours = 195, family/local = 147). This increased the model fit to χ^2^(278) = 2,915, CFI = 0.870, RMSEA = 0.057, 90% CI [0.055, .059], SRMR = 0.058. In a second step, we added four covariances between the trust items (modification indices: London/young = 319, young/old = 193, neighbours/old = 189, London/neighbours = 114). This increased the model fit to χ^2^(274) = 2,371, CFI = 0.897, RMSEA = 0.051, 90% CI [0.049, .053], SRMR = 0.056. In a final step, we added covariances between the two items measuring appropriateness of restrictions (MI = 121), and between the latent factors of political trust and trust in others (MI = 111). Since the remaining modifications suggested did not make much theoretical sense, we stopped the iterative process there.

^7^
We ensured of this with a series of MANOVA comparing each integration and cohesion area (IA) taken separately to the other areas average (Wilks’ Lambda test). Results showed significant differences in each case, IA1: *F*(8, 1,630) = 9.41, *p* < .001, η^2^_p_ = .044; IA2: *F*(8, 1,607) = 17.36, *p* < .001, η^2^_p_ = .080; IA3: *F*(8, 1,631) = 10.81, *p* < .001, η^2^_p_ = .050; IA4: *F*(8, 1,679) = 6.25, *p* < .001, η^2^_p_ = .029; IA5: *F*(8, 1,641) = 15.28, *p* < .001, η^2^_p_ = .069; IA6: *F*(8, 1,616) = 3.96, *p* < .001, η^2^_p_ = .019.
