# Supplementary material for: The social cohesion investment: Communities that invested in integration programmes are showing greater social cohesion in the midst of the COVID‐19 pandemic
Source: J Community Appl Soc Psychol. 2021 Apr 5;32(3):536–54. doi: 10.1002/casp.2522 (PMC8251431; doi:10.1002/casp.2522)
Supplement: Supplementary file 1 — Supporting Information [file CASP-32-536-s001.docx]

**Supplementary Material**

**SM1 – Demographics breakdown by sample, and comparison with official statistics**

|  | IA1 | | IA2 | | IA3 | | IA4 | | IA5 | | IA6 | | Kent | | Scotland | | Wales | |
| --- | --- | --- | --- | --- | --- | --- | --- | --- | --- | --- | --- | --- | --- | --- | --- | --- | --- | --- |
|  | Sample | Census | Sample | Census | Sample | Census | Sample | Census | Sample | Census | Sample | Census | Sample | Census | Sample | Census | Sample | Census |
| **Gender** |  |  |  |  |  |  |  |  |  |  |  |  |  |  |  |  |  |  |
| Male | 34.2 | 48.9 | 26.6 | 49.9 | 31.1 | 49.2 | 29.9 | 49.1 | 28.2 | 49.9 | 35.1 | 49.5 | 44.7 | 48.9 | 49.2 | 48.5 | 42.2 | 49.1 |
| Female | 65.8 | 51.1 | 73.4 | 50.1 | 68.9 | 50.8 | 70.1 | 50.9 | 71.8 | 50.1 | 64.9 | 50.5 | 55.3 | 51.1 | 50.8 | 51.5 | 57.8 | 50.9 |
| **Age** |  |  |  |  |  |  |  |  |  |  |  |  |  |  |  |  |  |  |
| 18-44 | 20.9 | 46.0 | 66.3 | 52.6 | 55.2 | 52.7 | 43.7 | 47.8 | 25.0 | 61.4 | 48.8 | 53.2 | 22.0 | 45.0 | 40.3 | 46.5 | 33.8 | 45.1 |
| 45-64 | 47.8 | 34.1 | 28.1 | 30.6 | 31.8 | 30.0 | 44.1 | 30.7 | 51.4 | 26.0 | 39.3 | 29.6 | 41.7 | 32.8 | 34.3 | 33.2 | 41.1 | 32.5 |
| 65+ | 31.3 | 19.8 | 5.6 | 16.8 | 13.0 | 17.3 | 12.2 | 21.5 | 23.6 | 12.6 | 11.9 | 17.2 | 36.4 | 22.2 | 25.4 | 20.3 | 25.1 | 22.4 |
| **Ethnicity** |  |  |  |  |  |  |  |  |  |  |  |  |  |  |  |  |  |  |
| White | 87.6 | 89.6 | 81.5 | 69.1 | 70.1 | 67.4 | 81.6 | 78.8 | 74.5 | 52.1 | 84.5 | 82.2 | 88.9 | 93.4 | 90.7 | 95.9 | 92.4 | 95.5 |

*Notes*. IA stands for the Integration and cohesion Areas, numbered from 1 to 6. “Sample” shows the present data. “Census” shows official data from the 2011 UK Census (Office for National Statistics; <https://www.ons.gov.uk/census>).

**SM2 – Structural Equation Modelling: Model and Specifications**

The following code was used to specify and run the SEM model on R (package *lavaan*).

**SEM model**

fit.mod <- '

# measurement model

poltrust =~ poltrust1 + poltrust2 + poltrust3

appro2 =~ appropri_guidelines + appropri_school

trust =~ trust_general + trust_london + trust_neigh + trust_young + trust_old

rela =~ connec_family + connec_friend + connec_colleag + connec_neigh + connec_local

wb =~ wellbeing1 + wellbeing2

op =~ optimism1 + optimism2

# regression equations

poltrust ~ regions_vs_LA + genderMF + Zage + whether_white + Zincome + Zstatusladder + Zpolorient

appro2 ~ regions_vs_LA + genderMF + Zage + whether_white + Zincome + Zstatusladder + Zpolorient

thermo_migrants ~ regions_vs_LA + genderMF + Zage + whether_white + Zincome + Zstatusladder + Zpolorient

nb_collac ~ regions_vs_LA + genderMF + Zage + whether_white + Zincome + Zstatusladder + Zpolorient

trust ~ regions_vs_LA + genderMF + Zage + whether_white + Zincome + Zstatusladder + Zpolorient

rela ~ regions_vs_LA + genderMF + Zage + whether_white + Zincome + Zstatusladder + Zpolorient

wb ~ poltrust + appro2 + thermo_migrants + nb_collac + trust + rela

op ~ poltrust + appro2 + thermo_migrants + nb_collac + trust + rela

# covariances

connec_neigh ~~ connec_local

connec_family ~~ connec_friend

connec_friend ~~ connec_colleag

connec_friend ~~ connec_neigh

connec_family ~~ connec_local

trust_london ~~ trust_young

trust_young ~~ trust_old

trust_neigh ~~ trust_old

trust_neigh ~~ trust_london

appropri_guidelines ~~ appropri_school

poltrustT ~~ trust

‘

**Analysis specifications**

fit <- sem(fit.mod, missing = “ML”, fixed.x = FALSE, data = data)

**SM3 – Complete Results of the Structural Equation Modeling Analyses**

Measurement model

|  | *b* | *SE* | *z*-value | *p*-value | Standardised estimate |
| --- | --- | --- | --- | --- | --- |
| Political trust =~ | | | | | |
| poltrust1 | 1.000 |  |  |  | .649 |
| poltrust2 | 1.122 | .068 | 16.45 | .000 | .678 |
| poltrust3 | -0.690 | .045 | -15.18 | .000 | -.380 |
| Appropriateness of restrictions =~ | | | | | |
| appropri1 | 1.000 |  |  |  | .261 |
| appropri2 | 1.937 | .140 | 13.86 | .000 | .343 |
| Trust in other people =~ | | | | | |
| trust1 | 1.000 |  |  |  | .872 |
| trust2 | 0.791 | .026 | 30.18 | .000 | .670 |
| trust3 | 0.758 | .028 | 27.44 | .000 | .625 |
| trust4 | 0.660 | .024 | 27.44 | .000 | .592 |
| trust5 | .541 | .026 | 20.62 | .000 | .429 |
| Density of relationships =~ | | | | | |
| rela1 | 1.000 |  |  |  | .622 |
| rela2 | 0.908 | .066 | 13.68 | .000 | .613 |
| rela3 | 0.593 | .064 | 9.25 | .000 | .399 |
| rela4 | 0.556 | .062 | 8.961 | .000 | .430 |
| rela5 | 0.685 | .064 | 10.70 | .000 | .533 |
| Subjective wellbeing =~ | | | | | |
| wellb1 | 1.000 |  |  |  | .918 |
| wellb2 | 0.948 | .020 | 48.03 | .000 | .910 |
| Optimism =~ | | | | | |
| opti1 | 1.000 |  |  |  | .780 |
| opti2 | 1.227 | .035 | 34.61 | .000 | .938 |

Regressions

|  | *b* | *SE* | *z*-value | *p*-value | Standardised  estimate |
| --- | --- | --- | --- | --- | --- |
| Political trust ~ | | | | | |
| Area | -.064 | .016 | -3.94 | .000 | -.095 |
| Gender | -.008 | .016 | -0.51 | .61 | -.013 |
| Age | -.085 | .016 | -5.15 | .000 | -.129 |
| Ethnicity | .018 | .029 | 0.62 | .54 | .015 |
| Income | -.057 | .018 | -3.18 | .001 | -.086 |
| Subjective status | -.127 | .018 | -7.26 | .000 | -.194 |
| Political orientation | -.060 | .015 | -3.86 | .000 | -.091 |
| Appropriateness of restrictions ~ | | | | | |
| Area | .039 | .009 | 4.13 | .000 | .170 |
| Gender | -.017 | .009 | -1.91 | .056 | -.075 |
| Age | .052 | .009 | 5.89 | .000 | .235 |
| Ethnicity | -.042 | .016 | -2.67 | .008 | -.104 |
| Income | .025 | .009 | 2.63 | .009 | .111 |
| Subjective status | .099 | .011 | 8.96 | .000 | .443 |
| Political orientation | .147 | .014 | 10.37 | .000 | .661 |
| Immigration attitudes ~ | | | | | |
| Area | .979 | .409 | 2.39 | .017 | .043 |
| Gender | 1.505 | .408 | 3.69 | .000 | .065 |
| Age | -.949 | .419 | -2.27 | .023 | -.042 |
| Ethnicity | -4.245 | .733 | -5.80 | .000 | -.105 |
| Income | 1.460 | .454 | 3.22 | .001 | .065 |
| Subjective status | 1.261 | .436 | 2.89 | .004 | .056 |
| Political orientation | -7.898 | .392 | -20.17 | .000 | -.351 |
| Social activism ~ | | | | | |
| Area | .492 | .043 | 11.52 | .000 | .208 |
| Gender | .051 | .043 | 1.19 | .23 | .021 |
| Age | -.028 | .044 | -0.63 | .53 | -.012 |
| Ethnicity | -.188 | .077 | -2.46 | .014 | -.045 |
| Income | .085 | .048 | 1.78 | .075 | .037 |
| Subjective status | .139 | .046 | 3.02 | .003 | .060 |
| Political orientation | -.594 | .041 | -14.51 | .000 | -.257 |
| Trust in other people ~ | | | | | |
| Area | .083 | .017 | 4.75 | .000 | .099 |
| Gender | .003 | .017 | 0.16 | .88 | .003 |
| Age | .175 | .018 | 9.75 | .000 | .214 |
| Ethnicity | -.048 | .031 | -1.55 | .122 | -.033 |
| Income | .052 | .019 | 2.69 | .007 | .064 |
| Subjective status | .077 | .019 | 4.15 | .000 | .095 |
| Political orientation | .009 | .017 | 0.52 | .61 | .011 |
| Density of relationships ~ | | | | | |
| Area | .092 | .020 | 4.59 | .000 | .114 |
| Gender | .056 | .020 | 2.81 | .005 | .069 |
| Age | -.117 | .022 | -5.42 | .000 | -.148 |
| Ethnicity | -.079 | .037 | -2.14 | .032 | -.056 |
| Income | .066 | .023 | 2.88 | .004 | .084 |
| Subjective status | .042 | .022 | 1.93 | .054 | .054 |
| Political orientation | -.001 | .019 | -0.04 | .98 | -.001 |
|  |  |  |  |  |  |
| Subjective wellbeing ~ | | | | | |
| Political trust | -.122 | .037 | -3.30 | .001 | -.089 |
| Appropriateness of restri. | 1.221 | .140 | 8.71 | .000 | .304 |
| Immigration attitudes | .004 | .001 | 5.46 | .000 | .109 |
| Social activism | .011 | .007 | 1.48 | .138 | .028 |
| Trust in other people | .150 | .025 | 5.92 | .000 | .137 |
| Density of relationships | .101 | .029 | 3.48 | .001 | .089 |
| Optimism for the future ~ | | | | | |
| Political trust | -.096 | .034 | -2.84 | .005 | -.076 |
| Appropriateness of restri. | 1.175 | .126 | 9.34 | .000 | .319 |
| Immigration attitudes | .002 | .001 | 3.05 | .002 | .061 |
| Social activism | .026 | .007 | 3.76 | .000 | .072 |
| Trust in other people | .174 | .024 | 7.27 | .000 | .173 |
| Density of relationships | .043 | .025 | 1.73 | .085 | .042 |

Covariances

|  | *b* | *SE* | *z*-value | *p*-value | Standardised estimate |
| --- | --- | --- | --- | --- | --- |
| rela4 ~~ rela5 | .427 | .028 | 15.01 | .000 | .543 |
| rela1 ~~ rela2 | .236 | .051 | 4.62 | .000 | .260 |
| rela2 ~~ rela3 | .227 | .034 | 6.67 | .000 | .230 |
| rela2 ~~ rela4 | .019 | .021 | 0.90 | .37 | .023 |
| rela1 ~~ rela5 | .072 | .029 | 2.52 | .012 | .085 |
| trust2 ~~ trust4 | .150 | .014 | 10.63 | .000 | .285 |
| trust4 ~~ trust5 | .120 | .013 | 9.41 | .000 | .175 |
| trust3 ~~ trust5 | .160 | .017 | 9.31 | .000 | .222 |
| trust2 ~~ trust3 | .049 | .012 | 3.93 | .000 | .088 |
| appropri1 ~~  appropri2 | .418 | .024 | 17.50 | .000 | .430 |
| Political trust ~~  Trust in others | .141 | .014 | 10.37 | .000 | .288 |

Explained variance

|  | *R*^2^ |  |  | *R*^2^ |
| --- | --- | --- | --- | --- |
| *Items* |  |  | *Latent variables* |  |
| poltrust1 | .421 |  | Political trust | .099 |
| poltrust2 | .460 |  | Appropriateness of restri. | .890 |
| poltrust3 | .145 |  | Immigration attitudes | .176 |
| appropri1 | .068 |  | Social activism | .147 |
| appropri2 | .118 |  | Trust in other people | .072 |
| trust1 | .760 |  | Density of relationships | .076 |
| trust2 | .449 |  | Wellbeing | .160 |
| trust3 | .390 |  | Optimism | .175 |
| trust4 | .351 |  |  |  |
| trust5 | .184 |  |  |  |
| rela1 | .387 |  |  |  |
| rela2 | .376 |  |  |  |
| rela3 | .159 |  |  |  |
| rela4 | .185 |  |  |  |
| rela5 | .284 |  |  |  |
| wellb1 | .843 |  |  |  |
| wellb2 | .828 |  |  |  |
| opti1 | .608 |  |  |  |
| opti2 | .880 |  |  |  |

**SM4 – Descriptive Statistics of Social Cohesion Measures for Each of the Six Integration Areas and Three Other Areas**

*Social Cohesion at the Macro Level*

*Notes*. IA stands for the Integration and Cohesion Areas, numbered from 1 to 6. IA6 is the one integration area that was identified as faring less well on the different indicators considered than the five others. ENG = the English county of Kent. SCOT = Scotland. Error bars represent standard error of the mean.

*Social Cohesion at the Meso Level*

*Social Cohesion at the Micro Level*

*Outcomes of Social Cohesion*
